# Longitudinal changes in brain connectivity correlate with neuropsychological testing in brain tumor resection patients

**DOI:** 10.3389/fnins.2025.1532433

**Published:** 2025-03-24

**Authors:** David G. Ellis, Matthew Garlinghouse, David E. Warren, Michele R. Aizenberg

**Affiliations:** ^1^Department of Neurosurgery, University of Nebraska Medical Center, Omaha, NE, United States; ^2^Nebraska-Western Iowa Veteran’s Affairs Medical Center, Omaha, NE, United States; ^3^Department of Neurological Sciences, University of Nebraska Medical Center, Omaha, NE, United States

**Keywords:** brain connectivity, brain tumor, graph theory, connectome, neuropsychological evaluation

## Abstract

**Background:**

Patients undergoing brain tumor resection experience neurological and cognitive (i.e., neurocognitive) changes reflected in altered performance on neuropsychological tests. These changes can be difficult to explain or predict. Brain connectivity, measured with neuroimaging, offers one potential model for examining these changes. In this study, we evaluated whether longitudinal changes in brain connectivity correlated with changes in neurocognitive abilities in patients before and after brain tumor resection.

**Methods:**

Patients underwent functional and diffusion MR scanning and neuropsychological evaluation before tumor resection followed by repeat scanning and evaluation 2 weeks post-resection. Using this functional and diffusion imaging data, we measured changes in the topology of the functional and structural networks. From the neuropsychological testing scores, we derived a composite score that described a patient’s overall level of neurocognitive functioning. We then used a multiple linear regression model to test whether structural and functional connectivity measures were correlated with changes in composite scores.

**Results:**

Multiple linear regression on 21 subjects showed that functional connectivity changes were highly correlated with changes in neuropsychological evaluation scores (R^2^ adjusted = 0.79, *p* < 0.001). Changes in functional local efficiency (*p* < 0.001) and global efficiency (*p* < 0.05) were inversely correlated with changes in composite score, while changes in modularity (*p* < 0.01) as well as the patient’s age (*p* < 0.05) were directly correlated with changes in composite score.

**Conclusion:**

Short interval changes in brain functional connectivity markers were strongly correlated with changes in the composite neuropsychological test scores in brain tumor resection patients. Our findings support the need for further exploration of brain connectivity as a biomarker relevant to brain tumor patients.

## Introduction

For brain tumor patients, surgical resection can increase longevity and enhance quality of life. These benefits are maximized when neurosurgeons exercise skill to avoid highly disruptive, surgically-induced deficits by using tools such as preoperative functional imaging and intraoperative stimulation (Luna et al., 2021; Hamer et al., 2012; Ellis et al., 2020). However, even when neurosurgeons appropriately utilize tools and expertise, many brain tumor patients still experience unexpected changes in their neurological and cognitive functioning (i.e., neurocognition) following surgery (Lacroix et al., 2001; Gulati et al., 2011; Jakola et al., 2011).

One potential means for exploring the relationship between changes in neurocognitive function related to observable changes in the brain could be to use brain connectivity markers derived from neuroimaging. Previous research has indicated the utility of using brain connectivity to better understand the effects of brain tumors and their treatment. For instance, functional and structural connectivity have been shown to have a significant impact on brain tumor patient outcomes (Salvalaggio et al., 2024). Interestingly, structural connectivity helps explain glioma infiltration patterns, and the disruption of the structural connectome beyond the focal lesion has been shown to impact survival (Wei et al., 2023). Meanwhile, functional connectivity has been shown to be altered in regions both proximal and distal to gliomas, and the quantity of these abnormal connections relates to tumor aggressiveness and cognitive function (Stoecklein et al., 2020). Furthermore, pre-surgical functional connectivity has been shown to have utility for predicting patient survival and functional status (Luckett et al., 2024; Luckett et al., 2023).

While brain connectivity has proven useful in understanding the effects of tumors on brain function, it has not yet been shown whether longitudinal changes in connectivity correlate to changes in neurocognition. Understanding the relationship between connectivity and neurocognition could inform the discovery of biomarkers that are relevant for non-invasive patient monitoring and surgical planning. To this end, we examined the relationship between graph network connectivity and neuropsychological measures in patients before and after tumor resection surgery and identified key connectivity markers predictive of cognitive and neurological changes.

## Methods

### Subject enrollment and clinical care

For this pilot study, adult patients (≥19 years old in Nebraska) were considered for enrollment if they had a supratentorial primary or metastatic tumor or cavernoma for which resective surgery was recommended. Subjects could not have had any prior brain treatments (surgery, radiation) or a history of a neurodegenerative disorder. After consent and enrollment, patients had preoperative clinical, neuropsychological, quality of life, and imaging (MRI) evaluations within 1 week prior to surgery. Tumor resection was performed via craniotomy for resection of their lesion, and the patients received standard perioperative clinical care. Two weeks postoperatively, clinical, neuropsychological, quality of life, and imaging studies were repeated. Healthy control subjects were also enrolled to evaluate the effect of repeat testing. For control subjects, no surgery was performed, but the same neuropsychological, quality of life, and imaging assessments were performed 2 weeks apart.

### Neuropsychological testing

Subjects and controls were administered neuropsychological evaluations and quality of life (QOL) inventories (Supplementary Table S1). This test battery was designed to assess cognitive and neurological functions commonly noted in the literature to be compromised in patients with gliomas (Johnson et al., 2012; Noll et al., 2015; Wefel et al., 2016). Testing domains consisted of basic attention, dexterity, executive, language, memory, and speeded processing. See Supplementary material for more details.

The total correct or raw scores for each test were converted to percentiles based on normative distributions provided by the test publisher. To assess the patient’s abilities within a given domain (listed in Supplementary Table S1), the reported percentile scores of the tests within each domain were averaged, similar to previous studies (Johnson et al., 2012; Armstrong et al., 2011; Wefel et al., 2011). We computed a single clinical trial battery composite (CTB Comp) score per subject from the averaged domain scores. We used this score to assess the overall combined changes in neurocognitive functioning and impairment per subject.

### Image acquisition

In addition to our standard clinical brain tumor MRI protocol at the scanning session visits mentioned above, we acquired research sequences consisting of high angular resolution diffusion MRI (dMRI) and 26 min of high-resolution resting-state functional MRI (rs-fMRI) according to the protocol from the Human Connectome Project on Development and Aging (HCP D/A; Harms et al., 2018). We used the Siemens Prisma 3 T MR scanner at the University of Nebraska Medical Center Core for Advanced Magnetic Resonance Imaging Facility (RRID:SCR_022468) for all scanning sessions. The HCP D/A designed the protocol for the Siemens Prisma scanner to optimize data quality and efficiency for developing and aging cohorts (Harms et al., 2018). This protocol allows for high resolution, 1.5 mm and 2 mm isotropic for dMRI and rs-fMRI, respectively. We acquired the dMRI data (TR = 3.23 s) with two shells, 1,500 and 3,000 s/mm^2^, with 92–93 directions per shell, each acquired twice in opposite phase encoding directions and 28 b0 volumes interspersed equally. In addition, we acquired a total of 1952 rs-fMRI volumes over four runs for a total of about 26 min of rs-fMRI data (TR = 0.8 s). Acquiring a large number of volumes over multiple runs has been shown to provide enhanced results for mapping functional connectivity in individual subjects (Finn et al., 2015; Pannunzi et al., 2017).

### Image processing

We performed image processing using an in-house processing pipeline written utilizing NiPype (Esteban et al., 2020) and incorporating processing workflows from fMRIPrep (Esteban et al., 2019a) and related projects (Esteban et al., 2019b). We designed the in-house pipeline to allow for enhanced customizability of the image registrations and transformations not offered in fMRIPrep. The node definitions were defined by the Schaefer et al. 300 parcellation seven-network atlas (Schaefer et al., 2018) in FSL’s asymmetric MNI space (Evans et al., 2012) as acquired from TemplateFlow (Ciric et al., 2010). To account for any distortions caused by surgery or tumor growth, the registrations between the preoperative and postoperative T1w scans for an individual patient were computed using non-linear registrations. All non-linear registrations were performed using the Advanced Normalization Tools (ANTs) SyN registration algorithm (Avants et al., 2009).

### Functional image processing

Head motion correction (Jenkinson et al., 2002) and susceptibility distortion correction (Andersson et al., 2003) were performed on the rs-fMRI using FSL (Woolrich et al., 2009) and fMRIPrep (Esteban et al., 2019a). The alignment between each rs-fMRI scan and the T1w image for that scanning visit was computed using a boundary-based rigid registration in FreeSurfer (Greve and Fischl, 2009). Transformation into MNI space through preoperative T1w space was performed in a single step that included head motion and susceptibility distortion correction transforms. Due to the TR being much shorter than standard fMRI sequences (TR = 770 ms for the rs-fMRI scans compared to a TR of about 2.5 s for a standard fMRI scan), we did not perform slice timing correction, which is the same approach used by the HCP for their processing pipelines (Glasser et al., 2013). To correct for artifacts in the BOLD acquisition, we adopted the Power et al. approach to denoising by simultaneously applying high-pass and low-pass filters, regressing out of 24 motion regressors along with global signal, and censoring of high motion timepoints (Power et al., 2014). Any scanning sessions with less than 5 min of resting state data following denoising were excluded from the analysis.

Following preprocessing, the whole brain functional networks were constructed with Nilearn (Abraham et al., 2014). The regions of interest from the Schaefer et al. parcellation atlas (Schaefer et al., 2018) were used as the nodes of the network (Aerts et al., 2018; Cheng et al., 2015), with the connections between nodes being defined as the temporal correlation between the regions of interest. To allow consistent comparison between scanning sessions, the networks were normalized to only include the connections with correlations at or above the 80th percentile (i.e., the network density was set at 20%).

### Diffusion image processing

The diffusion imaging data were processed in the native diffusion space. The alignment between the diffusion imaging and the T1w image for that scanning visit was computed using a rigid registration. Additionally, the diffusion data was corrected for head motion (Jenkinson et al., 2002), susceptibility distortions (Andersson et al., 2003), and eddy current distortions (Woolrich et al., 2009; Andersson and Sotiropoulos, 2016). Multi-shell multi-tissue constrained spherical deconvolution was used to estimate fiber orientation distributions (Jeurissen et al., 2014). Next, anatomically constrained tractography (ACT) was performed to generate white matter tracts for each subject and session (Smith et al., 2012). This method of tractography limits the white matter tracts to terminate mainly at the boundary between the gray matter and the white matter or within the deep gray. Constraining the tractography in this way makes the assignment of tracts to cortical regions straightforward. Finally, we applied spherical deconvolution-informed filtering of tractograms (SIFT) to the white matter tracts to filter out tracts less likely to be accurate (Smith et al., 2013). We then constructed the structural connectome matrix by counting the number of estimated white matter tracts between any two brain regions as defined by the Harvard-Oxford atlas transformed through the preoperative T1w space (Schaefer et al., 2018). Two nodes of the atlas were determined to be connected if five or more reconstructed tracts connect those regions. This overall method of reconstructing white matter tracts increases the accuracy of the tractography results (Jeurissen et al., 2019) and has been used previously to estimate the structural connectome in brain tumor patients (Aerts et al., 2018).

### Graph network measures

With both the functional and structural networks constructed, we computed graph network measures for all scanning visits. We focused on whole-brain network measures rather than individual nodes or connections due to the variability in the location of the tumors, tumor-induced brain disruptions, and surgical treatment. We focused on the following network measures that have shown promise in previous brain imaging studies: modularity, clustering coefficient, and global/local efficiency.

Modularity measures how well networks can be divided into modules. A module is a subset of nodes that are more densely connected to each other than to the rest of the network. A network with higher modularity will have modules containing nodes that are more closely connected to each other and more loosely connected to the nodes of other modules (Newman, 2006; Sun et al., 2014). Similarly, the clustering coefficient is a measure of the degree to which brain regions in the network tend to form tightly interconnected clusters or communities. Modularity of the functional brain network has been shown to be increased in early-onset multiple sclerosis (MS) patients and correlated negatively with task performance in those patients (Gamboa et al., 2014). The modularity of the functional network has also been shown to change in the brains of subjects undergoing sleep deprivation as well as those recovering from stroke (Ben Simon et al., 2017; Siegel et al., 2018). To compute the modularity of the brain networks, we assigned each node to a module based on the network assigned by Yeo et al. 7-network atlas (visual, somatomotor, dorsal attention, ventral attention, limbic, frontoparietal, and default mode; Schaefer et al., 2018; Yeo et al., 2011).

In addition to modularity and clustering coefficient, we also measured the global efficiency (i.e., the efficiency of the parallel information transfer in the network) as well as the mean local efficiency across all nodes (i.e., the fault tolerance of the network; Latora and Marchiori, 2001). All graph network connectivity measures were computed using the Brain Connectivity Toolbox for Python^1^ (Rubinov and Sporns, 2010).

**Figure 1:**
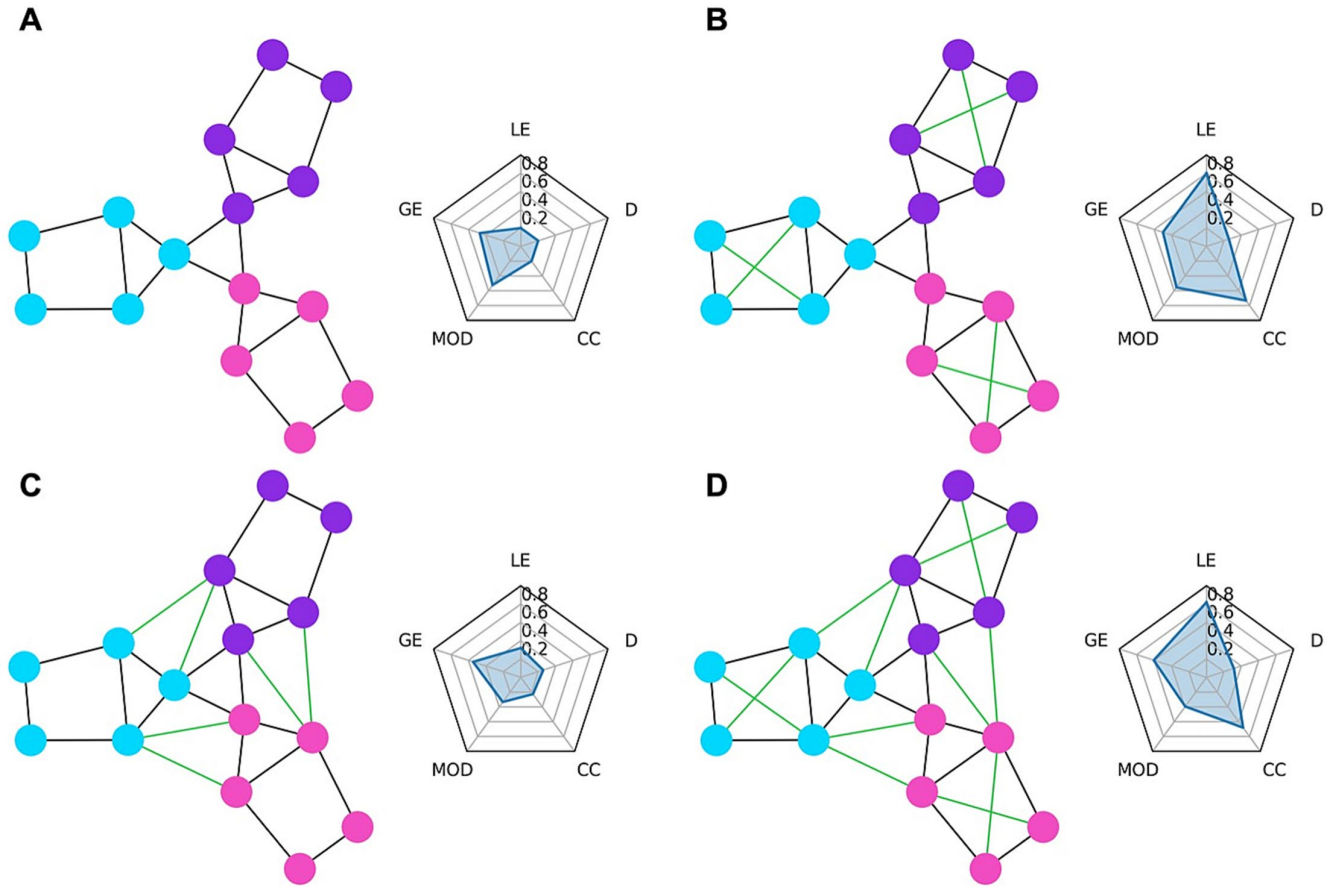
Example graph networks and corresponding graph theory measures shown in radar charts. The circles represent the nodes of the network while the lines represent the edges. Each node belongs to either the purple, blue, or pink module. The radar chart to the right of each network shows the graph theory measures of local efficiency (LE), global efficiency (GE), modularity (MOD), clustering coefficient (CC), and density (D) for that network. **(A)** Shows a simple network with three distinct modules. **(B)** Shows the network from **(A)** but with added connections (green edges) within each module. These within-module connections greatly increase the local efficiency (local fault tolerance) and clustering coefficient and slightly increase the modularity and global efficiency. **(C)** Shows the network from **(A)** but with added connections between modules which decrease modularity but provide a small increase to local efficiency. **(D)** Shows the network from **(A)** but with both the within module connections from **(B)** and between module connections from **(C)** added. Compared to **(B)**, the modularity is decreased due to the between module connections, while compared to **(C)** the local efficiency, global efficiency, and clustering coefficient are all increased due to the added within module connections.

In Figure 1, we show some examples of simple graph networks and how connections between and within modules change the network measures. Figure 1B shows that adding intra-module connections to the simple network, shown in Figure 1A, increases the clustering coefficient by making the modules form tighter network clusters and local efficiency by making the neighbors of many of the nodes more fault tolerant to loss of any given node. Figure 1C shows that adding inter-module connections decreases the modularity of the network by making each of the modules less segregated. Figure 1D shows that adding both inter- and intra-module connections produces a combination of decreased modularity with increased efficiency and clustering coefficient.

### Statistical analysis

To evaluate the relationship between brain connectivity measures and changes in neuropsychological assessments, we fitted a multiple regression linear model. We used both the structural and functional connectivity changes along with the demographic variables of sex and age as the predictor variables and the composite score changes as the response variable. Before fitting the model, we first removed redundant predictors by removing variables that had a Pearson correlation of absolute value greater than 0.8 to other predictors. We then performed feature selection using LASSO linear regression using glmnet in R (Friedman et al., 2010; Team, R.C., 2022). The selected features from the LASSO regression were used as the predictor variables to the multiple regression model evaluating the relationship between the predictors and the composite score changes.

## Results

### Enrollment

We enrolled a total of 38 patients, 21 of whom had complete sets of neuropsychological testing and MRI (Supplementary Table S2). As shown in Table 1, the average age at the time of surgery was 50.8 years (SD = 11.8). Table 2 shows the distribution of tumor diagnosis and tumor location: 51% of the cases were either high- or low-grade gliomas, and the cases were almost evenly split between the right (51%) and left (49%) hemispheres. After censoring timepoints affected by motion, each scanning session contained 12–26 min of resting state fMRI data (mean = 23.7 min, standard deviation = 4.2 min). The average number of days between scanning sessions was 18.2 days for patients and 18.7 for controls. To evaluate the effect of repeat testing, seven healthy control subjects were also enrolled, with 6 completing all evaluations. While the demographics of the control group were substantially different from that of the patient group in terms of age and education, the use of the control data was limited to the evaluation of changes in test scores following surgery and did not affect any of the other analyses.

**Table 1:** Subject demographics.

	Patients enrolled (*n* = 37)	Patients complete (*n* = 21)	Controls (*n* = 6)
Age (years)			
Mean (**±**SD)	50.1 (±11.8)	49.2 (±9.9)	32.8 (±3.8)
Range	26–71	33–64	27–37
Handedness			
R (%)	33 (89%)	18 (86%)	6 (100%)
L (%)	4 (11%)	3 (14%)	0 (0%)
Education (years)			
Mean (**±**SD)	13.8 (±2.3)	14.5 (±2.4)	19.2 (±1.8)
Range	11–18	11–18	16–21
Sex			
M (%)	24 (65%)	16 (76%)	3 (50%)
F (%)	13 (35%)	5 (24%)	3 (50%)

**Table 2:** Patients’ tumor characteristics.

	Enrolled (%)	Complete (%)
Classification		
LGG	5 (14%)	4 (19%)
HGG	14 (38%)	10 (48%)
Met	11 (30%)	3 (14%)
Meningioma	4 (11%)	3 (14%)
Cavernoma	3 (8%)	1 (5%)
Hemisphere		
R	18 (49%)	11 (52%)
L	19 (51%)	10 (48%)
Location		
Frontal	10 (27%)	6 (29%)
Frontoparietal	1 (3%)	0 (0%)
Occipital	4 (11%)	0 (0%)
Parietal	9 (24%)	6 (29%)
Temporal	11 (30%)	7 (33%)
Frontal/Cingulate	1 (3%)	1 (5%)
Insula	1 (3%)	1 (5%)

### Changes in neuropsychological test scores following surgery

**Figure 2:**
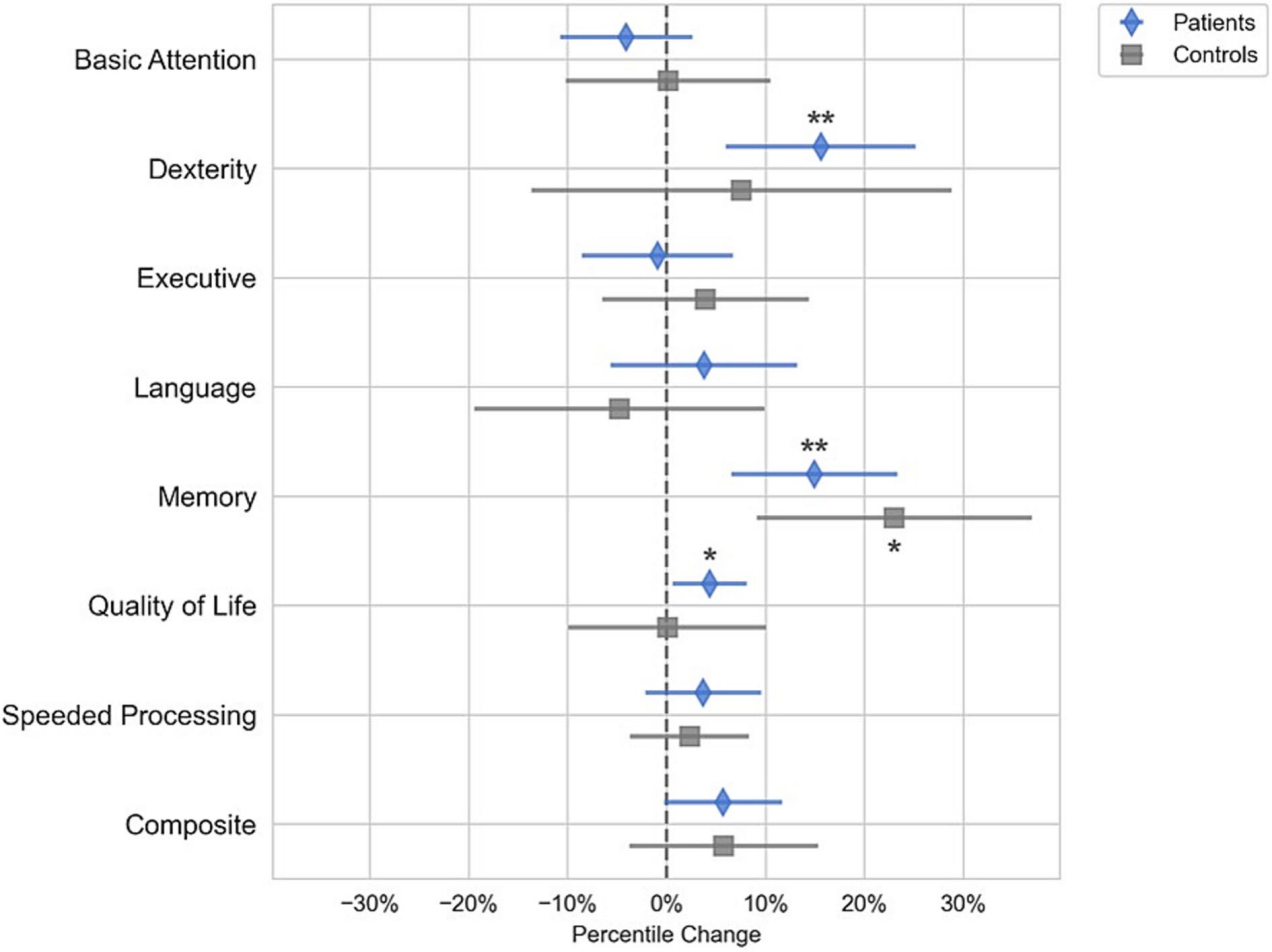
Changes in composite scores in brain tumor patients after tumor resection (blue diamond) and healthy controls (gray square). Quality of Life improved post-surgery (*p* < 0.05), as did Dexterity, and Memory scores (*p* < 0.005). However, these changes were not significantly different than those in the control group of healthy subjects that did not have surgery. Therefore, the improvements in Dexterity and Memory are potentially the result of practice increasing both the control and patient scores rather than surgery which would only increase the patient scores. All other cognitive domain scores did not statistically change from baseline in either the tumor or control cohort. (*p < 0.05, **p < 0.01).

The domain scores for Quality of Life, Dexterity, and Memory improved in patients postoperatively (*p* < 0.05), but these changes were not significantly different from the controls (Figure 2). The control group had a significant increase in the Memory domain scores (*p* < 0.05) but not for any of the other domains. Because none of the neuropsychological domain score changes for the patient group were different from the controls, we could not conclude that the neuropsychological domain scores changed because of surgery or tumor removal. Further analysis on the subset of patients that had gliomas showed that these patients followed the same trends as that of the entire cohort and had significant increases in memory and dexterity scores following surgery.

### Feature selection

We removed the changes in mean functional clustering coefficient from the analysis because it strongly correlated with changes in functional global efficiency, with Pearson r = −0.91 (Supplementary Figure S2). All features were z-score normalized and LASSO regression was used to select the best features. The LASSO regularization weight was optimized using 10-fold cross validation. The regularization weight resulting in the lowest validation mean-squared error corresponded to a validation of R^2^ = 0.55, meaning that 55% of the variance in the composite scores were explained by the predictor variables. This regularization weight was used to train a final LASSO model to choose the best predictor variables. This model eliminated sex, structural modularity, and structural global efficiency from the analysis. The remaining six features were used to fit a multiple regression model without regularization on the 21 patients with complete neuropsychological testing and MRI.

### Multiple linear regression analysis

**Figure 3:**
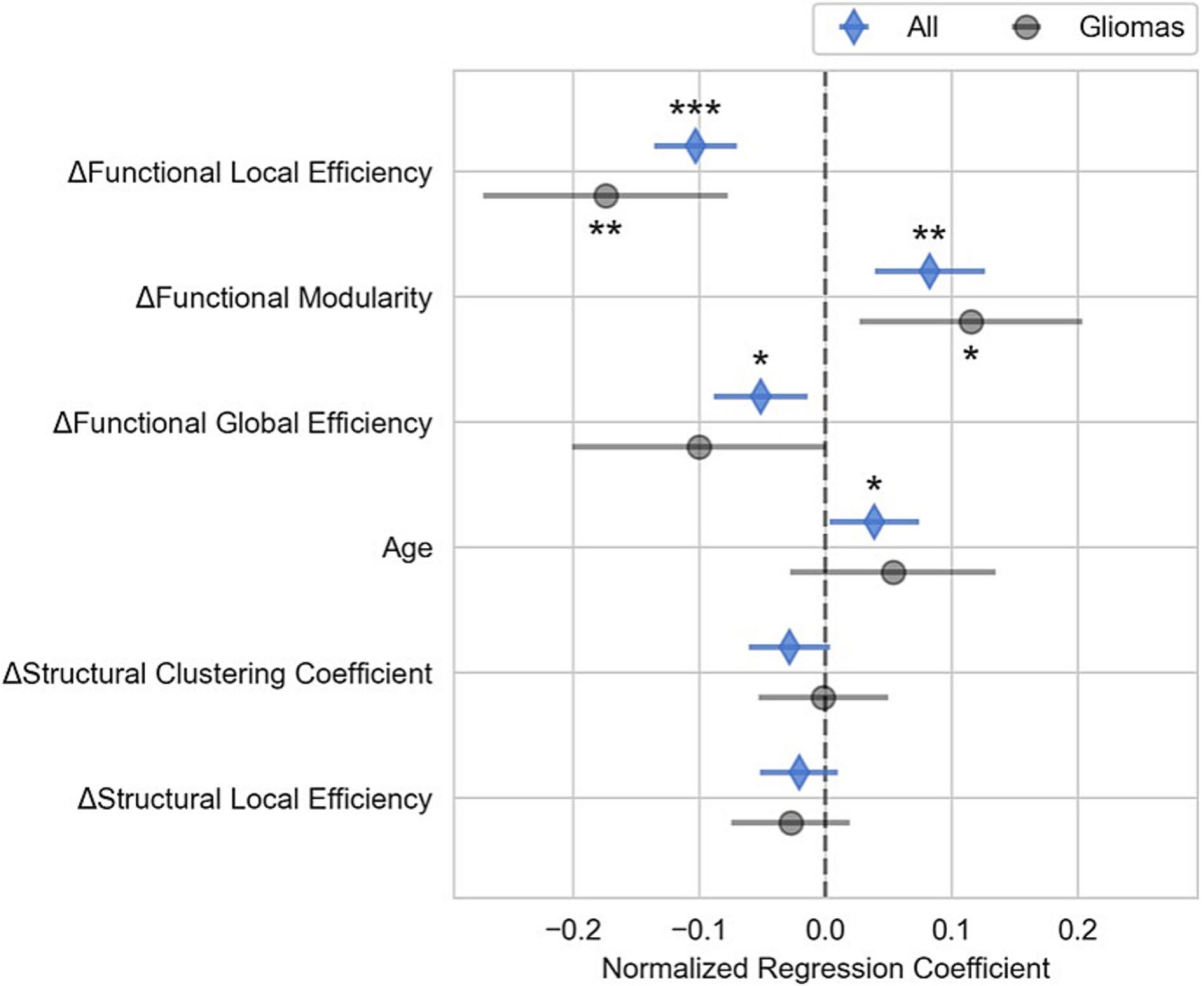
Relationship between changes in composite neuropsychological test scores and in brain connectivity for brain tumor resection patients. This figure shows the normalized regression estimates (sorted by *p* value) and 95% confidence intervals of the multiple linear regression model predicting changes in the composite neuropsychological score for all patients (blue diamond) as well as the subset of glioma patients (gray circles). Changes in functional local and global efficiency were inversely correlated with changes in composite score while changes in functional modularity and age were directly correlated with changes composite score. The gliomas subset shows that the relationship between connectivity markers and composite score for patients with infiltrative tumors is similar to that of the whole cohort. (*p < 0.05, **p < 0.01, ***p < 0.001).

The multiple regression model showed that changes in connectivity was highly correlated with changes in the neuropsychological composite scores (R^2^ = 0.85, R^2^ adjusted = 0.79, F-statistic = 13.4, *p* < 0.001). Changes in functional local efficiency (*p* < 0.001), functional modularity (*p* < 0.01), and functional global efficiency (*p* < 0.05) as well as the patient’s age (*p* < 0.05) were significantly correlated with changes in composite neuropsychological score, as shown in Figure 3. Functional local efficiency and modularity demonstrated the strongest associations with composite score, and these associations were maintained when examining only the subset of patients with gliomas (Figure 3). Specifically, functional local efficiency was inversely correlated with composite score while functional modularity was directly correlated.

We also fitted additional multiple linear regression models with each of the domain scores, including quality of life, as the outcome variable to assess if the relationship between connectivity varied by domain. As shown in Supplementary Figure S3, we found that the relationship between the domain scores and the connectivity variables followed the same trend as that of the main model.

## Discussion

This study shows that changes in functional brain network connectivity were highly correlated with neuropsychologic measure changes in brain tumor resection patients. Specifically, our model revealed a strong relationship between neuropsychological test score changes and changes in the functional brain connectivity measures of local and global efficiency as well as modularity.

The finding that functional modularity was directly correlated with neuropsychological measures corroborates previous studies showing functional modularity to be a biomarker associated with improved cognitive functioning (Siegel et al., 2018; Alexander-Bloch et al., 2010; Gallen and D’Esposito, 2019; Jalili, 2017). For instance, Siegel et al. found significantly increased functional modularity at 3 months post-stroke in patients with good recovery from language, spatial memory, and attention deficits (Siegel et al., 2018).

The strong inverse relationship between changes in mean functional local efficiency and neuropsychological testing scores suggests that increasing functional local efficiency may have negative effects on neurocognitive functioning in brain tumor patients. Other research studies have found functional local efficiency to be negatively correlated with cognitive performance (Stanley et al., 2015; Kawagoe et al., 2017; Cohen and D'Esposito, 2016). In a study of 29 healthy adults, Stanley et al. found that functional local efficiency during working memory tasks was inversely correlated to working memory performance (Stanley et al., 2015). This finding supports the role of decreased local efficiency correlating to better cognitive performance. Interestingly, Stanley et al. only found the local efficiency to be predictive of working memory performance during task performance and not while the subject was at rest (Stanley et al., 2015), while our results show that the changes to the local efficiency at rest are highly predictive of overall changes to neuropsychological measures. Also supporting the inverse role of functional local efficiency in cognitive performance, Kawagoe et al. performed a cross-sectional study in elderly individuals and found that higher functional local efficiency at rest correlated to lower executive function performance and worse physical fitness (Kawagoe et al., 2017). While it is not clear as to why better functional local efficiency would negatively affect neurocognitive functioning, one explanation could be that increased hyper-local integration is a sign of adaptation to surgical insult. We hope that future research will further elucidate how the brain connectivity of tumor patients relates to their neurocognitive functioning.

While functional connectivity measures correlated to changes in composite score, we did not see an overall change in the composite neuropsychological score following surgery relative to the controls. Dexterity and memory functioning scores improved; however, these improvements were not significantly different from the control group. Because both controls and patients improved in their performance on these assessments, it is likely that the improvement in these domains represents the improvement due to practice effects rather than surgical treatment. Quality of life metrics improved postoperatively in the surgery group, indicating that tumor resection had a positive impact on patients’ well-being. Similar to the composite scores, changes in functional local efficiency and functional modularity were significant predictors of changes in quality of life (Supplementary Figure S3).

Structural connectivity changes were not correlated to neuropsychological measures in our study. This finding may result from several different hypotheses or a combination of them. First, our methodology for measuring structural connectome may not be sensitive to variable yet localized changes in white matter connections. A more sensitive marker for observing structural connectome changes may be fractional anisotropy. Second, structural connections may be best explored by looking at integrity of specific tracts rather than at network level descriptors such as modularity and local efficiency. Third, our methodology of anatomically constrained tractography may be ill suited for tumor patients due to disruption of normal anatomy. Fourth, in our patient cohort, structural connectivity may be more related to tumor mass effect rather than changes in the underlying pathology over this short interval. Lastly, structural connections may be relatively unperturbed because of surgical resection.

Tumor resections inevitably involve white matter in addition to gray matter. Tractography, which we are not visualizing here, may be affected by resection depending upon location of the resection and the importance of the tract functionality. There may be some changes to the tracts that may not translate into neurologic dysfunction. Changes in connectivity are possibly a better means of assessment. We know that neurologic function can have “collateral” paths such that an injury in an area will not result in an overt neurologic deficit. This may occur due to connectivity changes such that other areas or structures are “picking up the slack.” Structural and functional connectivity changes likely occur in tandem and enable the brain to continue to function optimally in circumstances of injury (stroke, tumor, surgery, injury, etc.).

## Limitations

Our results serve as a preliminary analysis to test the utility of brain connectivity markers to explain changes in neuropsychological test scores and to identify key connectivity measures most predictive of neurocognitive outcomes in brain tumor resection patients. A crucial next step is to validate the predictive ability of these brain connectivity measures in an independent cohort of patients in a longitudinal study.

We modeled the brain connectivity measures together in a single multiple linear regression model rather than in separate models. Combining the connectivity measures into a single model is intuitive, as brain connectivity is complex and unlikely to be convincingly captured by a single metric. This approach, however, requires that the interpretation of the effects of a single brain connectivity metric be made with caution. The coefficients associated with each connectivity measure in the model represent the relationship between that specific measure and the changes in neuropsychological test scores while holding all other variables constant. *In situ*, however, brain connectivity measures do not change in isolation, and inferences about changes in neurocognitive scores can only be made when accounting for the changes in all the variables.

We observed low compliance from our patients for the neuropsychological testing, likely due to the mental demands of the neuropsychological evaluations under already stressful circumstances for the patients (Burke et al., 2019). Interestingly, compliance with MR scanning was much higher, indicating that, if robust and replicable associations are found, brain connectivity markers could be a less burdensome means of tracking cognitive and neurological functioning.

Even when patients with significant impairments complied with testing, many of the tests were not sensitive enough to measure changes in states of impairment. For example, an elderly patient in our study presented with language deficits and poor overall neurocognitive functioning. This patient was unable to complete most of the assessments both preoperatively and postoperatively preventing us from tracking any postoperative changes from baseline. However, upon clinical assessment, the physician (author MA) noted an improvement in their functioning. Therefore, this patient group may be better monitored with neuropsychological measurement tools that can detect changes in the levels of impairment without being overly burdensome.

Another factor may be timing. These assessments were conducted only 2 weeks apart, and it may be that the neuropsychological testing changes are transient and lack clinical relevance. In the postoperative period, patients experience the effects of medications, brain shift, physical fatigue, sleep deprivation, and other factors that may affect brain function. The amount of time necessary for the resolution of these changes and their effects is unknown. We selected our time interval for testing to isolate surgical effects as well as minimize perioperative medication effects.

## Conclusion

We found that short interval changes in brain connectivity markers were highly correlated with changes in the composite neuropsychological test scores. Our findings support the need for further exploration of brain connectivity as a biomarker relevant to the neurocognitive status of brain tumor resection patients. After further validation, brain connectivity markers might aid in tracking the effects of treatment on patient cognitive functioning, potentially reducing reliance on neuropsychological testing. Future research could also explore using anticipated changes in brain network topology to better inform surgical approaches. By modeling brain networks resulting from different tumor resection strategies prior to surgery, it may be possible to identify approaches that optimize brain network characteristics and improve patient outcomes. Furthermore, future research could also explore changes in brain connectivity that result from tumor interactions with neurons. Nonetheless, further research is needed to better understand how surgical and other interventions affect brain networks and how network changes impact neurocognitive outcomes.

## Data Availability

The raw data supporting the conclusions of this article will be made available by the authors by request.

## Footnotes

^1^ https://pypi.org/project/bctpy/
