# Supplementary material for: Longitudinal changes in brain connectivity correlate with neuropsychological testing in brain tumor resection patients
Source: Front Neurosci. 2025 Mar 24;19:1532433. doi: 10.3389/fnins.2025.1532433 (PMC11973353; doi:10.3389/fnins.2025.1532433)
Supplement: Supplementary file 1 [file Data_Sheet_1.docx]

Supplementary Material

**Neuropsychological Testing**

Neuropsychological tests included the verbal memory based on a list learning task (AVLT), visual memory for a complex geometric shape (RCFT), baseline neurocognitive ability (WRAT-IV), speeded processing/alternation (Trails A and B), verbal fluency (Controlled Oral Word Association Test), dexterity (Grooved Pegboard), general expressive and receptive language (Boston Naming Test, Sentence Repetition and NAB Auditory Comprehension Test), working memory (NAB Digits Forward and Backward; Wechsler Memory Scale, Third Edition Spatial Span Forward and Backward), and the ability to inhibit a pre-learned or automatic response (Stroop Color and Word Test). Additionally, this test battery includes measures of effort based on embedded MNB measures, including Forced Choice and patterns of recognition memory. Mood and quality of life were assessed via several questionnaires, including the MD Anderson Symptom Inventory – Brain Tumor (MDASI-BT) [1], Functional Assessment of Cancer Therapy – Brain (FACT-Br) [2], and Frontal Systems Behavior Scale (FrSBe)[3].

| **Domain** | **Neuropsychological Assessment** |
| --- | --- |
| **Basic Attention** | NAB Digits Forward [4] |
|  | NAB Orientation [4] |
|  | WMS-III Spatial Span Forward [5] |
| **Dexterity** | Grooved Pegboard [6] |
| **Executive** | HRB Trails B [6] |
|  | Stroop Inference [7] |
|  | NAB Digits Backward [4] |
|  | WMS-III Spatial Span Backward [5] |
| **Language** | Controlled Oral Word Association [6] |
|  | Boston Naming Test [6] |
| **Memory** | Auditory Verbal Learning Test [6] |
|  | Rey Complex Figure Test [6] |
| **Speeded processing** | HRB Trails A [6] |
|  | Stroop Color and Word [7] |
| **Quality of life (QOL)** | MDASI-BT [1] |
|  | FACT-Br [2] |
|  | FrSBe [3] |

***Table S1*** *- List of neuropsychological and QOL assessments for clinical evaluation. The composite score is calculated from the average of the normalized composite scores for each domain.*

**Clinical Assessment**

Objective clinical assessment was performed by the neurosurgery clinicians. Cognition, orientation, attention, fluency, and thought content were assessed. Further language functions were assessed by reading, writing, naming, repetition. Parietal function was assessed by testing or determining presence of: calculation, agraphesthesia, extinction, proprioception, neglect, apraxia. Motor function was assessed by strength testing and motor scale. Visual deficits were assessed by testing visual field quadrants to confrontation.

**Image Registration**

To minimize registration errors between the MNI template space and the patients’ T1w images, we performed the transformations between MNI and T1w space using the preoperative T1w image. In our experience, the registration between the MNI template and the preoperative T1w images has proven much more reliable than the registration between the MNI and the postoperative T1w images due to poor alignment of areas containing surgical defects. To work around registering between MNI and postoperative space, we computed the transforms between the postoperative T1w and the preoperative T1w images. We then concatenated these intra-subject transforms with the preoperative space to MNI transforms so that all transformations into and out of MNI space were performed through the preoperative space. In addition to minimizing registration errors, performing transformations through preoperative space ensured that the node definitions remained more consistent for an individual patient than if the registrations between MNI space were performed separately for each scanning session.

**Statistical Analysis**

To investigate the effect of surgery on neurocognitive performance, we computed the change for each neurocognitive domain and composite score. We then performed a one-sample t-test to determine if the changes were significantly different from zero. We also performed a two-sample t-test to determine if the changes in the neurocognitive scores of the patients were significantly different from those of the control group.

Supplementary Results

**Data Collection**

|  | **Preop** | **Postop** | **Both** |
| --- | --- | --- | --- |
| **MRI** | 37 | 34 | 34 |
| **Neuropsych** | 31 | 30 | 30 |
| **Complete Neuropsych** | 24 | 24 | 22 |
| **Complete Neuropsych + QOL** | 20 | 19 | 17 |
| **Complete MRI + Neuropsych** | 22 | 23 | 21 |
| **Complete MRI + Neuropsych + QOL** | 19 | 18 | 16 |

***Table S2*** *- Number of patients that completed MRI scanning and neuropsychological evaluation. While nearly all the 38 enrolled patients completed MRI scanning both preoperatively and postoperatively, many patients did not complete the neuropsychological and quality of life (QOL) exams.*

**Clinical Assessment**

At the time of surgery, 17 patients (46%) had pre-existing deficits. At the 2-week postoperative appointment, 4 of the patients with pre-existing deficits saw all their deficits resolve, 3 saw improvement to at least one of their deficits, 7 saw no change to their deficits, and 4 either saw their deficits worsen or developed new deficits. In addition, 4 of the patients with no pre-existing deficits had developed deficits by the 2-week postoperative appointment. However, 3 out of 4 of these patients with new deficits would see their deficits resolve at a later follow-up date.

|  | Pre-existing  (% of all patients) | Resolved | Improved | No change | Worsened | Unknown | New Deficits |
| --- | --- | --- | --- | --- | --- | --- | --- |
| Motor | 4 (11%) | 2 | 0 | 1 | 1 | 0 | 2 |
| Language | 5 (14%) | 2 | 0 | 3 | 0 | 0 | 3 |
| Sensory | 3 (8%) | 0 | 0 | 3 | 0 | 0 | 2 |
| Cognitive | 5 (14%) | 0 | 2 | 3 | 0 | 0 | 1 |
| Visual | 5 (14%) | 1 | 1 | 1 | 2 | 0 | 2 |

**Table S3** - Clinical deficits. Pre-existing deficits were noted at the preoperative visit and the effect of surgery on those deficits at the 2-week postoperative visit are noted as well as new deficits.

*
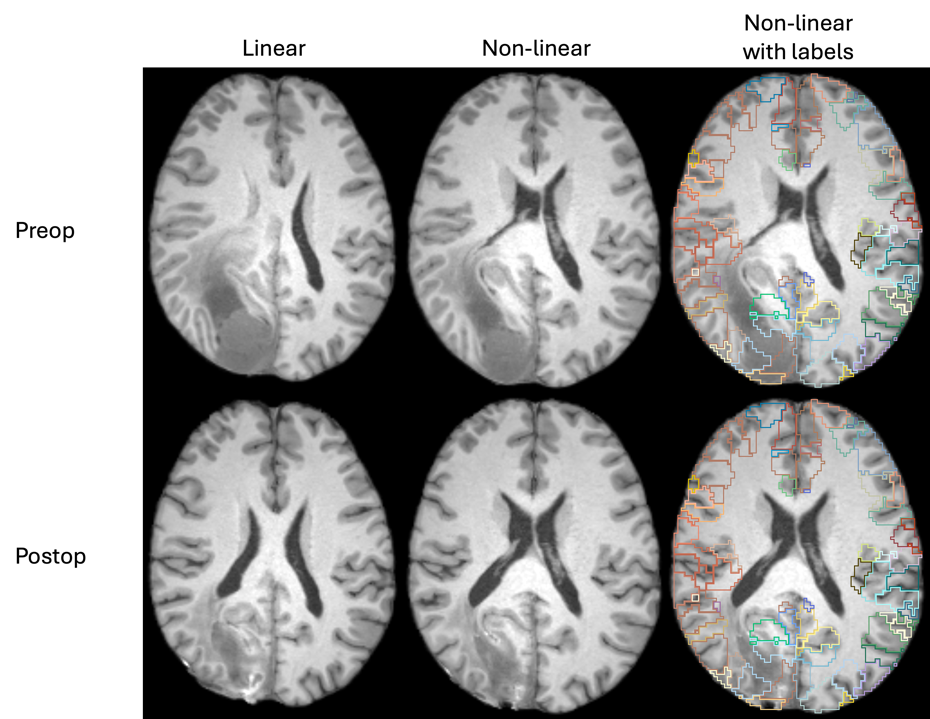
***Image Registration**

***Figure S1*** - The above figure shows the preoperative and postoperative T1w images in MNI space following non-linear postoperative to preoperative alignment. In the leftmost column, the images are aligned to the MNI template via a linear registration. In the middle column, the images are aligned to the MNI template via a non-linear registration as described in the methods section. The right column shows the label map used for the node definitions over the aligned images.

**Variable Selection**


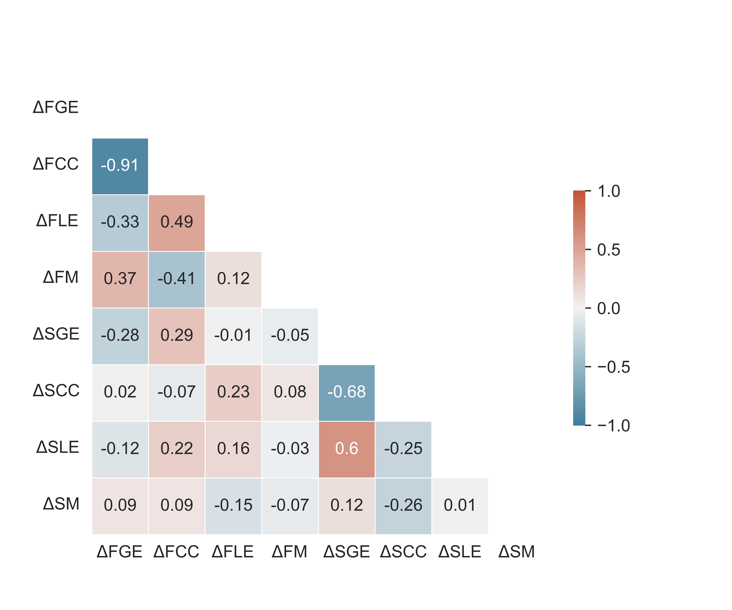


**Figure S2** - Correlation between changes in connectivity measures. Increases in functional clustering coefficient (FCC) were highly correlated with decreases in functional global efficiency (FGE). Due to the high correlation (pearson r > 0.8) between changes in FCC and changes in FGE, we excluded changes in FCC from the multiple linear regression model predicting changes in cognitive scores. All other variables had low enough correlation between themselves to be included in the regression model and analysis. (FGE=Functional Global Efficiency; FCC=Functional Clustering Coefficient; FLE=Functional Local Efficiency; FM=Functional Modularity; SGE=Structural Global Efficiency; SCC=Structural Clustering Coefficient; SLE=Structural Local Efficiency; SM=Structural Modularity)

**Domain-specific Multiple Linear Regression Analysis**

To investigate whether different neurocognitive domains correlate independently to the brain connectivity predictions, we performed additional multiple linear analyses, as shown in ***Figure S3***. The relationships between the predictor variables and the domain scores were similar to the relationships between predictors and composite score. Quality of life scores were not included in the composite score, but ***Figure S3*** shows that the relationship between quality of life and the predictor variables was similar to that of the composite score as well. The most significant predictor from the main model, functional local efficiency, had inverse relationships to all testing domains.


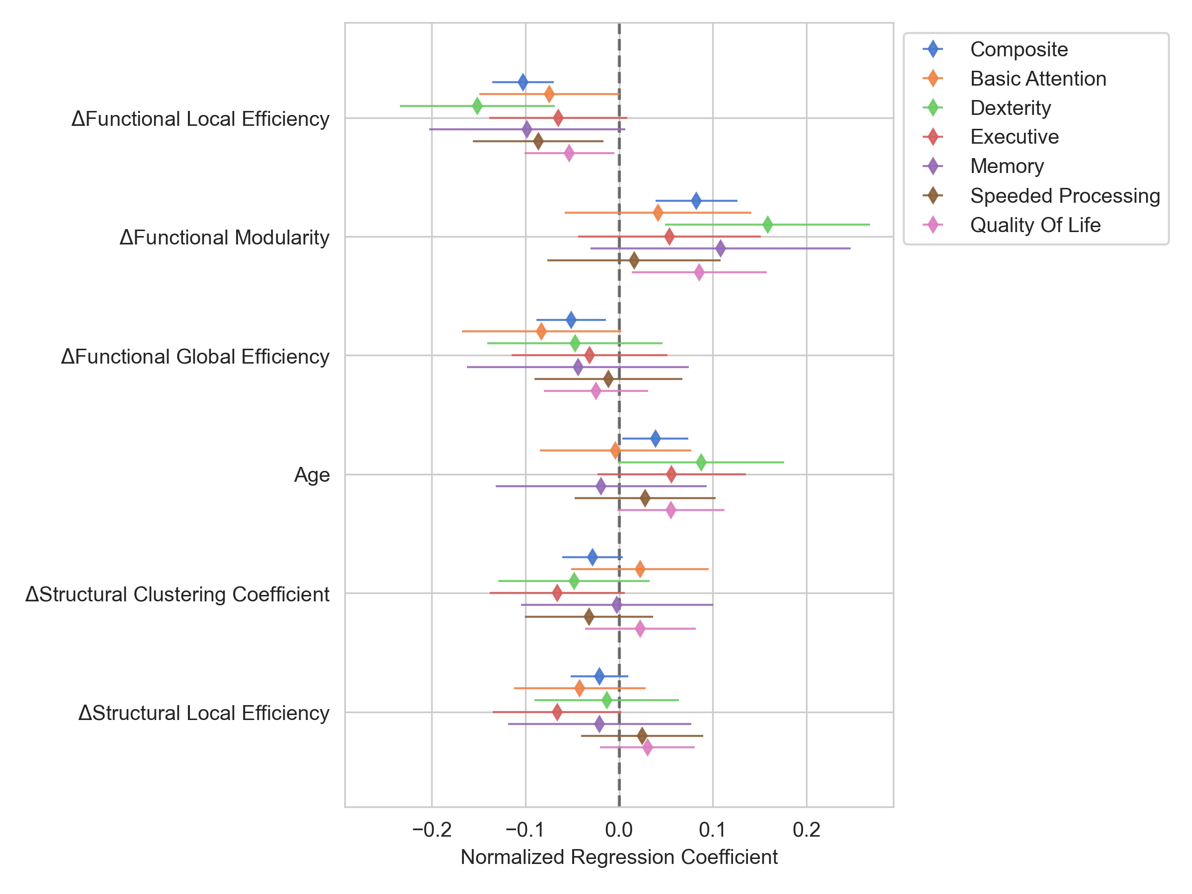


**Figure S3** - Normalized regression coefficients for multiple linear regression models describing the relationship between changes in connectivity and changes in cognitive scores for each domain including quality of life (QOL). The regression coefficients for each of the neuropsychological domains roughly follows the coefficients for the composite score model. The similarity between the coefficients for the individual domains and the composite model could indicate that the connectivity measures are detecting large scale brain changes that affect all of a patient’s cognitive abilities similarly. Of note, QOL was not included in the composite model, but the relationship between QOL and connectivity variables mirrors that of the composite model.

Simple Linear Regression Analysis

We also analyzed the predictive ability of changes in mean functional local efficiency and Functional Modularity to changes in composite using separate simple linear regression models, as shown in ***Figure S4***. Even in the absence of a multiple linear regression model, changes in mean functional local efficiency (left) demonstrate a strong inverse correlation, Pearson r = -0.77, p < 0.001, to changes in composite scores (***Figure S4A***). Changes in functional modularity did not show a significant correlation to changes in composite score, Pearson r = 0.10, p = 0.656 (***Figure S4B***). even though the relationship between these two variables was highly significant in the multiple regression model. However, changes in modularity did show a slightly positive correlation with the residuals from the mean functional local efficiency model, though this relationship did not reach statistical significance, Pearson r = 0.38, p = 0.086, (***Figure S4C***). Therefore, changes in modularity trend towards being predictive after accounting for changes in functional local efficiency and are only highly predictive after accounting for the other predictor variables in the main model.


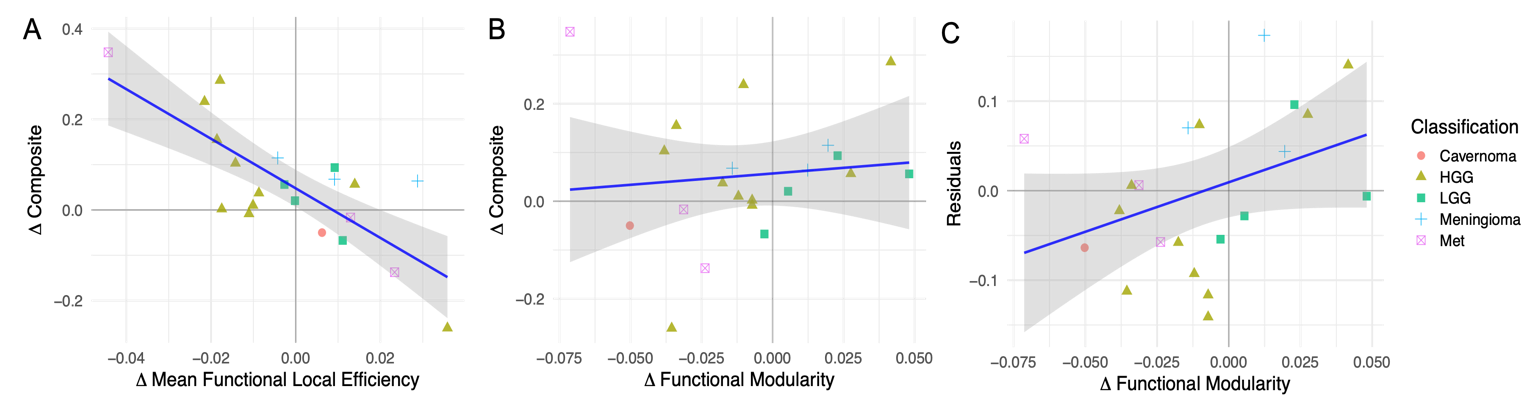


**Figure S4** - Simple linear regression results between composite score and top predictor variables. Even in the absence of a multiple linear regression model, changes in mean functional local efficiency (A) demonstrate a strong inverse correlation (Pearson r = -0.77, p < 0.001) to changes in composite scores. Changes in functional modularity (B) do not show a significant correlation to changes in composite score even though the relationship between these two variables was highly significant in the multiple regression model. Changes in modularity do show a slightly positive correlation with the residuals from the mean functional local efficiency model (C), though this relationship did not reach statistical significance (Pearson r = 0.38, p = 0.086). This indicates that the changes in modularity trend towards being predictive after accounting for changes in functional local efficiency and are only highly predictive after accounting for the other predictor variables in the main model.

**Multiple Linear Regression Using Preoperative Connectivity**

***Figure S5*** *- To assess the impact of preoperative connectivity on changes on the composite neuropsychological scores, we repeated the multiple linear regress using preoperative connectivity markers as the predictor variables instead of the changes in connectivity as was done previously. This resulted in no connectivity variables showing significant association with the composite score. For the model, the R^2^ was 0.44, adjusted R^2^ was -0.03, F-statistic = 0.94 with p value = 0.53.*


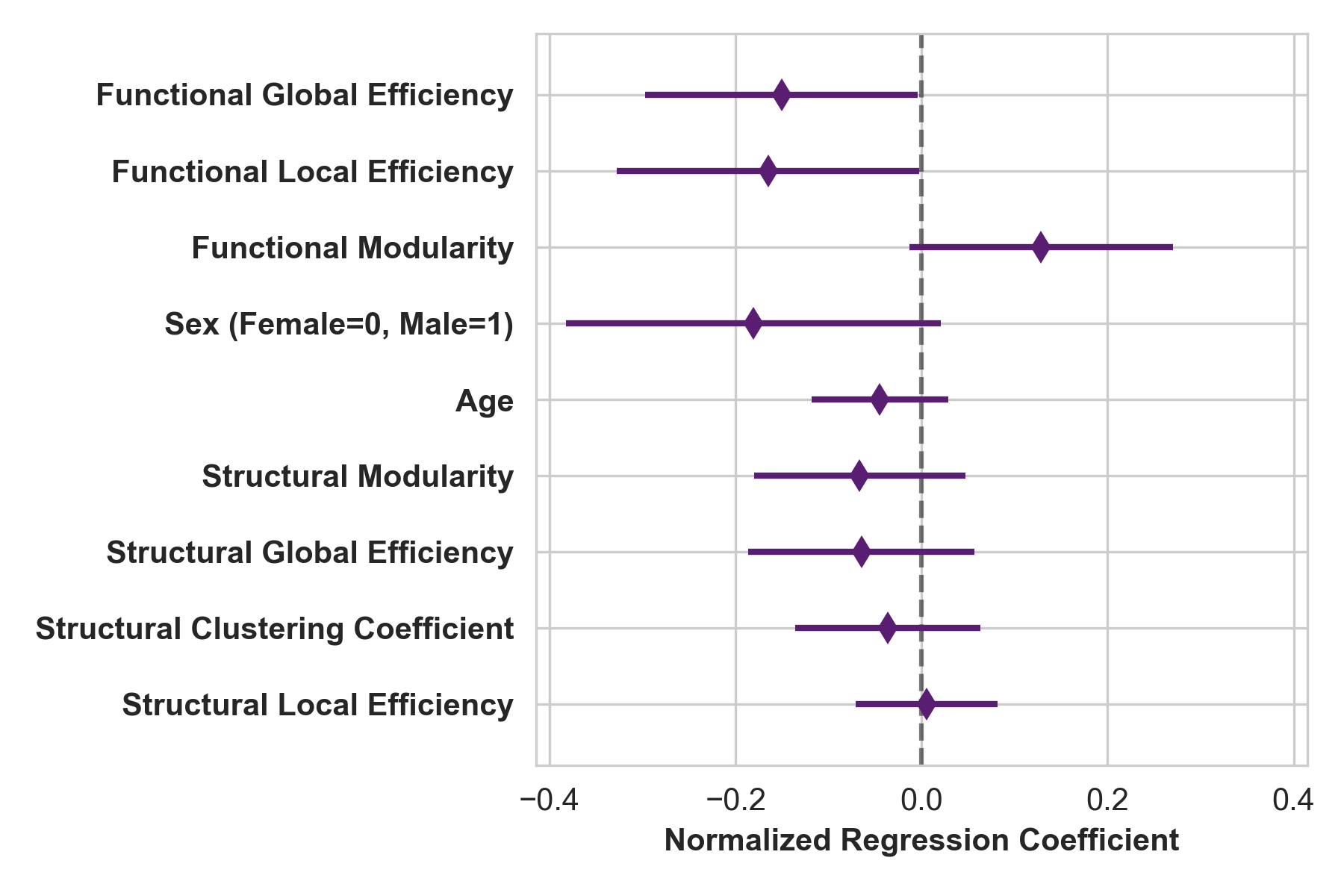


References

1. Armstrong, T.; Mendoza, T.; Gring, I.; Coco, C.; Cohen, M.; Eriksen, L.; Hsu, M.-A.; Gilbert, M.R.; Cleeland, C. Validation of the MD Anderson symptom inventory brain tumor module (MDASI-BT). *J. Neurooncol.* **2006**, *80*, 27-35.

2. Weitzner, M.A.; Meyers, C.A.; Gelke, C.K.; Byrne, K.S.; Levin, V.A.; Cella, D.F. The Functional Assessment of Cancer Therapy (FACT) scale. Development of a brain subscale and revalidation of the general version (FACT‐G) in patients with primary brain tumors. *Cancer* **1995**, *75*, 1151-1161.

3. Grace, J.; Malloy, P.H. *Frontal systems behavior scale (FrSBe): Professional manual*; Psychological Assessment Resources (PAR): 2001.

4. Iverson, G.L.; Brooks, B.L.; White, T.; Stern, R.A. Neuropsychological Assessment Battery: Introduction and advanced interpretation. **2008**.

5. Scale, W.D.W.M. WMS-III. *San Antonio: The Psychological Corporation* **1997**.

6. Meyers, J.E.; Volbrecht, M.; Axelrod, B.N.; Reinsch-Boothby, L. Embedded symptom validity tests and overall neuropsychological test performance. *Arch. Clin. Neuropsychol.* **2011**, *26*, 8-15.

7. Golden, C.; Freshwater, S. Stroop Color and Word Test Adult Version. *A manual for clinical and experimental uses.(2a ed.). Wood Dale, Illinois: Stoelting* **2002**.
